# Using vitality indicators to predict survival of aquatic animals released from fisheries

**DOI:** 10.1093/conphys/coae034

**Published:** 2024-05-31

**Authors:** R J Lennox, M R Donaldson, G D Raby, K V Cook, L LaRochelle, J C Madden, S J Cooke, D A Patterson, S G Hinch

**Affiliations:** Ocean Tracking Network, Department of Biology, Dalhousie University, Halifax, NS B3H 4R2, Canada; Pacific Salmon Ecology and Conservation Laboratory, Department of Forest and Conservation Sciences, University of British Columbia, 2424 Main Mall, Vancouver, BC V6T 1Z4, Canada; Fish Ecology and Conservation Physiology Laboratory, Department of Biology, Carleton University, 1125 Colonel By Dr., Ottawa, ON K1S 5B6, Canada; Department of Biology, Trent University, 2089 East Bank Drive, Peterborough, ON K9L 1Z8, Canada; InStream Fisheries Research, 1121A Enterprise Way, Squamish, BC V8B 0E8, Canada; Fish Ecology and Conservation Physiology Laboratory, Department of Biology, Carleton University, 1125 Colonel By Dr., Ottawa, ON K1S 5B6, Canada; Fish Ecology and Conservation Physiology Laboratory, Department of Biology, Carleton University, 1125 Colonel By Dr., Ottawa, ON K1S 5B6, Canada; Fish Ecology and Conservation Physiology Laboratory, Department of Biology, Carleton University, 1125 Colonel By Dr., Ottawa, ON K1S 5B6, Canada; Fisheries and Oceans Canada, Cooperative Resource Management Institute, School of Resource and Environmental Management, Simon Fraser University, Burnaby, BC V5A 1S6, Canada; Pacific Salmon Ecology and Conservation Laboratory, Department of Forest and Conservation Sciences, University of British Columbia, 2424 Main Mall, Vancouver, BC V6T 1Z4, Canada

**Keywords:** Bycatch, catch and release, fisheries, mortality, reflex indicators

## Abstract

Estimating the survival probability of animals released from fisheries can improve the overall understanding of animal biology with implications for fisheries management, conservation and animal welfare. Vitality indicators are simple visual measures of animal condition that change in response to stressors (like fisheries capture) and can be assessed to predict post-release survival. These indicators typically include immediate reflex responses which are typically combined into a score. Vitality indicators are straight-forward and non-invasive metrics that allow users to quantify how close (or far) an animal is from a normal, ‘healthy’ or baseline state, which in turn can be correlated with outcomes such as survival probability, given appropriate calibration. The literature on using vitality indicators to predict post-release survival of animals has grown rapidly over the past decade. We identified 136 papers that used vitality indicators in a fisheries context. These studies were primarily focused on marine and freshwater fishes, with a few examples using herptiles and crustaceans. The types of vitality indicators are diverse and sometimes taxa-specific (e.g. pinching leg of turtles, spraying water at nictitating membrane of sharks) with the most commonly used indicators being those that assess escape response or righting response given the vulnerability of animals when those reflexes are impaired. By presenting Pacific salmon fisheries as a case study, we propose a framework for using vitality indicators to predict survival across taxa and fisheries.

## Teaser

Simple vitality indicators are being used to assess fish health and condition following fisheries interactions. These indicators have been determined to be predictive of post-release survival for a number of taxa. There is much promise for the widespread application of these simple but effective tools.

## Introduction

The viability of live release as a conservation tactic in commercial and recreational fisheries depends on survival and reproductive fitness of the animals. Fishes (Broadhurst *et al.*, 2006), mammals (Read *et al.*, 2006), birds (Gales *et al.*, 1998), herptiles (Watson *et al.*, 2005) and crustaceans (Stoner, 2012) are caught as bycatch in large numbers, and incidental mortality has led to population declines for some species (Lewison *et al.*, 2004). Voluntary release of target or non-target species in recreational fisheries is analogous and may be practiced for a variety of ethical, cultural, legal or personal reasons (Arlinghaus *et al.*, 2007). As many as 30 billion individual fish may be captured and released annually by recreational fisheries alone (globally; Cooke and Cowx, 2006) with a larger but generally unknown number captured in commercial fisheries (Alverson *et al.*, 1994). Many released fish ultimately survive and reproduce, but that is not the case for all fish or fisheries interactions (Chopin and Arimoto, 1995; Campbell *et al.*, 2010a; Raby *et al.*, 2011; Wilson *et al.*, 2014). Animals that are released (without tracking devices) have unknown fate, and fisheries therefore rely on scientific validation of mortality predictors that can improve fishing practices and guide management approaches.

Efforts to understand and mitigate mortality associated with fisheries releases have yielded positive results (Davis, 2002) but there has long been a need for research that informs fisheries on how to assess the condition of animals upon release (Broadhurst *et al.*, 2009). Fisheries interactions can cause injury (ranging from dermal injuries to hook damage), physiological alterations (e.g. depletion of tissue energy stores, neuroendocrine stress response, elevation of heart rate), behavioural impairments (e.g. locomotor impairments) which can contribute to immediate or delayed mortality. Yet, there is need for rapid and easy-to-use measures of fish condition that provide insight into organismal status and are predictive of mortality. Davis (2010) proposed that use of vitality indicators may approximate mortality following appropriate calibrations of stressor testing with controls. Having fishers or fisheries observers rapidly assess fish vitality following capture could be a means of generating rapid and inexpensive estimates of post-release mortality (Davis, 2007, 2010) and could be used to compare the effects of different fishing methods (e.g. gear types or handling methods) or environmental conditions on fish survival. Physiological tools (e.g. measuring blood constituents) can be useful for predicting delayed mortality (Moyes *et al.*, 2006) but are expensive and can require time for interpretation, expertise for execution of the techniques and may themselves induce stress that affect outcomes (Cooke *et al.*, 2013; Sopinka *et al.***,** 2016). Conversely, vitality indicators, which can include the popular reflex action mortality predictors (RAMPs) as described by Davis (2010), are easy-to-use and inexpensive field-based assessments that measure fish condition before release to predict future survival. In this technique, reflexes are chosen (relying on stimuli such as light, gravity, sound and touch) where the response can be scored in a binary manner and combined together to represent a level of impairment and stress (Davis, 2010). Assessment of vitality is usually rapid (<20 s), and the results can be integrated into a simple index of the animal’s status. In addition to being used to generate context-specific numerical estimates of survival probability, vitality indicators could also be used to make decisions about individual fish, such as whether to retain, release or use special revival techniques (Farrell *et al.*, 2001; Donaldson *et al.*, 2013).

A landmark review by Davis (2010) provided guidance for using reflex impairment assessments via the introduction of RAMP. Researchers have applied and expanded on this foundation, including reflex and injury assessments specific to fisheries and contexts. Here, we provide a broad, updated review of the literature on fish vitality indicators, which have since diversified and grown to be used in various taxa and fishery contexts. One goal of this review was to synthesize what has been learned about context-specific strengths and limitations of vitality indicators as mortality predictors in fisheries. Next, we present a case study of how vitality indicators have been used in Pacific salmon (*Oncorhynchus* sp.) fisheries to better understand post-release survival and to refine fisheries practices across sectors. We conclude by proposing a decision framework to inform those considering the application of vitality indicators in fisheries.

## Materials and Methods

We conducted a literature survey to identify papers that used vitality indicators to assess post-release survival up to January 2021. Google Scholar and Web of Science were used to identify relevant papers across all taxa that used vitality indicators at the time of capture to assess fish condition and/or to predict post-release mortality. In Web of Science, the keyword search included the following terms: vitality indicators OR Reflex Action Mortality Predictors OR RAMP OR reflex impairment AND fish*. Additional searches in Google Scholar included various combinations of the following key words: *vitality indicators, reflex action mortality predictors, RAMP, reflex impairment, injury, ventilation rates, ventilation depth, sublethal effects, behavioural impairment* in combination with *release fisheries, catch-and-release, capture-and-release fisheries, bycatch, fisheries mortality.* To ensure that we captured injury and wounding as predictors of vitality, we searched Google Scholar with the following additional phrase: *vitality indicators injury wound predict survival fisheries*.

The keyword literature search using Web of Science returned 79 results which were exported to a spreadsheet. The Google Scholar search combinations yielded over 2000 papers, with more than 100 papers that appeared to be directly relevant to vitality indicators, fisheries and post-release survival. After non-research papers and duplicates were excluded, screening at title and abstract was undertaken to remove non-relevant papers. This resulted in 47 relevant papers. An additional 10 relevant papers were added to the spreadsheet through additional searches in Google Scholar and browsing those papers that cited key papers and review articles (e.g. Davis, 2010). Our literature survey was not designed to be exhaustive (i.e. not a systematic review), but to capture the vast majority of peer-reviewed journal articles that have used vitality indicators in a fisheries context.

In addition to bibliographic information, the following data were then extracted from each paper: *year of study, species, general taxon (e.g. fish, reptile, or crustacean), study location, marine vs freshwater, vitality indicators studied (i.e. injury, barotrauma, reflex, ventilation, condition index, activity), whether the individuals were held, how survival was assessed, sampling unit, survival monitoring duration (i.e. immediate, hours, day, weeks, months, years), whether or not the vitality indicators effectively predicted survival, and any other relevant covariates that were found.*

**Figure 1:**
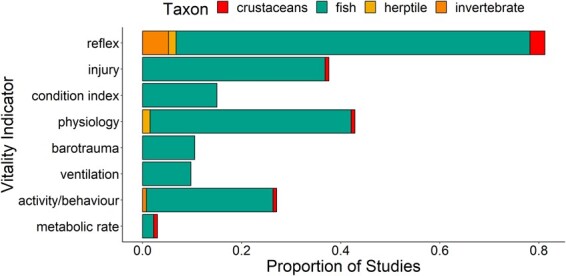
Studies for each of four taxa, crustaceans, fishes, herptiles and invertebrates, using different classes of vitality indicators for studying fisheries interactions among 136 studies identified in a structured review.

## Findings from evidence synthesis

### Overall results

This synthesis includes 136 studies that used vitality indicators in a fisheries context. The types of vitality indicators identified in the synthesis included reflex or behavioural/activity impairments, observable injury including signs of barotrauma and ventilation rates (Fig. 1). Reflex impairment assessments were the most common relative to the other types of vitality indicators. Reflex impairments and injury were also sometimes combined into an index or score commonly referred to as RAMP, although some studies used slightly different terminology, such as reflex impairment index or condition index. Of the studies identified here, 90 were carried out in the marine environment and 46 in freshwater. Seventy-seven of the 90 marine studies were focused on fishes whereas 10 examined crustaceans. In freshwater, 43 studies used fish and three focused on herptiles (i.e. turtles).

**Figure 2:**
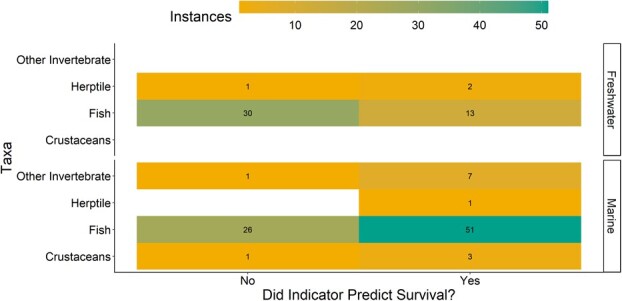
Results of the literature survey revealing the extent to which reflex indicators were predictive of survival (based on significance) relative to taxa.

Among the 136 studies identified in our synthesis, 98 tested whether there was a correlation between the vitality measure and survival (or mortality). Among these 98, 77 (79%) found a relationship between the vitality measures and survival, albeit across varying time intervals and using diverse methods for measuring the survival response (Fig. 2). Additionally, 78% of studies where individuals were held during or after the evaluation process found a correlation between vitality and survival, and similarly 82% of studies without any holding found a correlation with survival. Among studies where the testing was at an individual level, 89% found a correlation, while 70% of studies which tested at a grouped level (i.e. treatment) found a correlation with survival.

### Taxa-specific results

#### Fish

The majority (120 of the 136; 88%) of the studies reported using fish and a wide variety of fish taxa ranging from salmonids to sharks. Among fish, reflex indicators (see Table 1) were most frequently used, following the convention of Davis (2002); 84% of fish studies used such an index to test the effects of a stressor on vitality.

**Table 1:** Overview of vitality indicators and their strengths and weaknesses

Reflex	Previously used on	Description	Strength/Weakness	Key Reference(s)
Gag/Bite Response	Fish, crustaceans	A probe is inserted into the esophagus or mouth to test for involuntary muscle contractions that result in a gag or bite response	Test can be used for majority of taxa	Davis (2010)
Ventilation/Opercular response	Fish	Observing if the fish is actively attempting to ventilate (e.g. rhythmic opercular beats or movement of the lower jaw); Called opercular flaring in some papers	Only useful for fish with opercula that can be easily observed	Raby *et al.* (2012); Gale *et al.* (2014); McArley and Herbert (2014)
Dorsal spine response	Fish	Tested by moving a probe over the dorsal spines and observing if the spines erect	Only works with fin fish that have spines	Davis (2010)
Vestibular-ocular response (VOR)	Fish	Observed by turning the fish laterally to observe if the fishes eye tracks the with gravity	Tracking of the eyes only stops when the animal is essentially dead; Often used as an indicator that a fish is dead	Raby *et al.* (2012)
Equilibrium/righting response	Fish, turtles,	Turning the individual supine and determining if it can right itself (usually in 3 seconds); See Fig. 5	Does not work very well on large animals; Ecologically relevant in that a fish unable to maintain equilibrium would be susceptible to predation; Among the most common reflex tests	Stoner (2009); Donaldson *et al.* (2013); Stoot *et al.* (2013)
Escape response	Fish, turtles	Grasping the animal posteriorly (tail) to see if the animal tries to escape; Occasionally referred to as the burst response	Not possible with larger animals that are difficult to handle; Among the most common reflex tests	Brownscombe *et al.* (2013); Stoot *et al.* (2013)
Body flex response	Fish	Holding the fish out of water briefly to observe if the body muscles flex or by inserting a needle into the hypaxial musculature of the animal	Only possible on animals that can be held out of the water safely	Donaldson *et al.* (2013); Campbell *et al.* (2010a); McArley and Herbert (2014)
Nictitating membrane response	Sharks	Using a syringe, water is shot at the eye to determine if the nictitating membrane fires	Only useful for sharks with nictitating membranes	Gallagher *et al.* (2014)
Startle response	Turtles	Noise or movement of an object was presented and if the animal retracted their head, they perceived the threat	May only be useful for turtles but some possibility it could be applied to some invertebrates	Stoot *et al.* (2013)
Leg Response	Crustaceans, turtles	Leg flare (legs raised to near horizontal plane), leg retraction (legs retract when walking legs are drawn forward) and kick (one or more legs or chelipeds move quickly in the ventral direction)	Only useful for animals with arm/leg like appendages	Stoner (2009); Ledain *et al.* (2013); Rose *et al.* (2013)
Chela response	Crustaceans	Chelae open and close without manipulation	Only useful for crustaceans	Stoner (2009); Rose *et al.* (2013)

Forty-one percent of fish studies used an assessment of fish injury. For studies evaluating injury, the severity of the injury was typically ranked and integrated into a score by examining external injuries such as wounds to the body including gill and eye injuries, scale loss, mucous loss and fin damage (e.g. Nguyen *et al.*, 2014). Relevant covariates to mortality and vitality included total length (found in 6% of studies), depth of capture (8%), blood physiology (usually lactate; 6%), air exposure (8%), air temperature (3%) and water temperature (9%). Fourteen studies (12%) specifically focused on classifying barotrauma (Hannah *et al.*, 2014), which combines multiple vitality indicators to understand the effects of catching fish at depth and rapidly bringing them to the surface. For example, a unique study by Rogers *et al.* (2011) used an optokinetic reflex test to assess changes in visual performance of rosy rockfish (*Sebastes rosaceus*) that had experienced exophthalmia (i.e. protrusion of the eye associated with barotrauma). Rudershausen *et al.* (2014) investigated hook injury and barotrauma to estimate the discard mortality of reef fish. Additional observable behaviours have also been investigated, such as swimming ability observed with underwater video (Hannah and Jones, 2012).

#### Herptiles

Herptiles are frequently captured as bycatch in commercial fisheries targeting fish; however, there were only four instances in our review. Three of these four studies identified a correlation with condition. Additionally, sex, blood physiology and water temperature were all found to be relevant covariates in one of four studies. Bycatch of turtles is particularly urgent given their poor conservation status worldwide and therefore vitality indicators could be extremely useful. Bycatch reduction methods are commonly studied to reduce interactions between turtles and fishing gear, but tools are also needed to assess the condition of turtles that are captured. Vitality indicators have been validated for sea turtles (Ryder *et al.*, 2006) as well as freshwater eastern musk turtle (*Sternotherus odoratus*), northern map turtle (*Graptemys geographica*) and painted turtle (*Chrysemys picta*; Ledain *et al.*, 2013; Stoot *et al.*, 2013). Stoot *et al.* (2013) and Ledain *et al.* (2013) used six behavioural tests to monitor the post-capture condition of freshwater turtles including escape ability, righting ability (both on land and in water), startle-response (i.e. audible/pressure and visual) and tactile stimuli to the head, limbs and tail.

#### Crustaceans

Behavioural, reflex and injury responses have been examined as vitality indicators to assess post-release mortality in nine species of crustaceans and molluscs among 12 studies (Stoner, 2012). Species included snow crab (*Chionoecetes opilio*; Stoner *et al.,* 2008; Stoner, 2009; Urban, 2015), tanner crab (*Chionoecetes bairdi*; Stoner, 2009; Yochum *et al.*, 2015), hard clam (*Meretrix lusoria*; Lee *et al.,* 2007) and spot prawn (*Pandalus platyceros*; Stoner, 2012). No known freshwater crustaceans have been studied to date. Eleven of the 12 studies used reflexes and one study observed activity as a vitality indicator. Two studies found that size was a relevant covariate for vitality and mortality, while one found the same for sex and another for species. For crabs, Stoner (2009) examined righting behaviour, as well as a series of reflexes that were validated and later used by Rose *et al.* (2013); see Table 1).

### Synthesis

Vitality indicators have usually been found to be predictive of post-release mortality (79% predictive of studies that measured mortality) across a variety of fisheries for fish and invertebrates. These numbers were generated across a large variety of time scales from hours for studies following up immediately after capture to weeks or months using electronic tags to track fate or by holding animals in various captive facilities. Our review suggests that there is good potential for expanding the adoption of vitality indicators as part of standard fisheries monitoring and management initiatives for both commercial and recreational fisheries (Falco *et al.*, 2022). Importantly, our review suggests that these methods do not work for every species or in every context, so practitioners should be cautious about applying these tools without validation. Vitality indicators are easiest for smaller animals that can be handled safely, despite a number of studies assessing larger animals like adult sharks. Marine mammals that are often captured as bycatch will be challenging to adapt vitality indicators for. Context-specific differences, including among species (e.g. Depestele *et al.,* 2014) or seasons (e.g. Campbell *et al.,* 2010a), seem to be the most likely explanations for differences among studies, so validation studies are essential to determine if vitality indicators are effective at predicting survival for a particular combination of species and fishing gear. Results from a validation study could be used to justify applying vitality indicators on another closely related species or a different fishery context, but with much less certainty in the accuracy of any mortality estimate. Vitality indicators alone, if impaired, can be indicative of issues that require further examination and, in that sense, can be used to rapidly screen specific fisheries to assess the likelihood that mortality (or sublethal stress) may be problematic (Patterson *et al.*, 2017a).

### Methods and validations

Standard approaches for assessing post-release mortality in fisheries that release animals alive include containment experiments (i.e. use of tanks or net pens) and telemetry-tracking. Using those standard approaches to assess survival rates in the hundreds of potential species × gear-type × location × environmental condition combinations for which fisheries managers desire estimates of survival would be impractical. Indeed, this is the reason that interest in vitality assessments has grown so rapidly in recent years: vitality assessments can be used in fisheries observer programs as a proximate means to estimate rates of survival at the individual and grouped level. For example, in Canada’s Atlantic fisheries, vitality assessments have been used to quantify discard survival in a variety of commercially relevant fishes (Benoit *et al.,* 2010, 2012). In Europe, recently imposed restrictions on discarding has motivated renewed interest in discard fate because the policy allows for discarding to continue in the event that survival potential can be shown to be high (Uhlmann *et al.*, 2016). We expect vitality assessments will become an increasingly common tool in fisheries management in the coming years, as fisheries managers and researchers strive to understand and quantify survival potential in released fish and other aquatic animals (Breen and Catchpole*,* 2021).

Species-specific responses to stimuli determine the extent to which vitality indices are consistent indicators of animal condition. Vitality indices are therefore not recommended to be compared among species and require specific validations. Two types of validation exist: (i) proximate validation in which the mechanisms of action that control reflex or vitality response are tested against physiological responses to understand how specific indicators reflect the underlying condition of an animal; and (ii) ultimate validation in which an index is rigorously tested against outcomes of interest, generally mortality, without considering why or how the vitality indicators provide information so long as they do so reliably. Where possible, the goal should be to have mechanistic understanding and predictable outcomes. Proximate validations are rare and require physiological tools for studying oxygen transport to tissues and the brain, muscle acidosis, neuronal control of the musculature and the autonomic nervous system that regulates breathing/gill ventilation; Davis (2010) and Raby *et al.* (2012) provide some discussion of the physiological basis for some reflex assessment tests in fish. Validation can occur in the field, but a number of validation studies have taken place under controlled conditions in the lab or as part of a combined lab and field validation study. For example, Uhlmann *et al.* (2016) took a multi-faceted approach by validating reflex indicators in the lab for European plaice (*Pleuronectes platessa*) and common sole (*Solea solea*) before applying the RAMP to predict mortality following commercial trawling in a field setting. Davis and Ottmar (2006) examined injury and reflex impairment to predict mortality following simulated net capture in the laboratory. In that study, walleye pollock were more sensitive to injury from the net simulations, which also corresponded with mortality, but this was not the case for the other species examined. However, reflex impairment was significantly related to mortality in walleye pollock (*Gadus chalcogrammus*), sablefish (*Anoplopoma fimbria*), northern rock sole (*Lepidopsetta polyxystra*), and Pacific halibut (*Hippoglossus stenolepis*). Field-based validations have also been conducted using telemetry as a means of determining mortality. For example, Raby *et al.* (2012) conducted a validation study on coho salmon and found that radio-tagged individuals with greater reflex impairment had higher post-release mortality. Likewise, Yergey *et al.* (2012) used acoustic telemetry to link vitality indicators with delayed mortality for summer flounder (*Paralichthys dentatus*) caught in a commercial trawl fishery.

There are two reflex indicators that are relatively easy to use and in general tend to be among the most responsive indicators—the equilibrium and bursting indicators. Moreover, these are among the easiest to assess (in all but the largest of animals). This is not to say that there is not value in other reflex tools (especially across diverse taxa), but these have great ecological relevance (e.g. if an animal cannot maintain equilibrium, it will be unable to engage in directed movement, if an animal does not burst away when stimulated by a human it reveals an impairment that could lead to post release predation). We certainly encourage more work on reflex assessment but based on the literature reviewed here and our experiences with using these indicators on a wide range of species and in diverse contexts, those two indicators repeatedly rise to the top. Regardless, when building vitality-survival curves, the recommended use of 5–8 reflexes by Davis (2010) seems to remain good practice (Humborstad *et al.,* 2016; Meeremans *et al.,* 2017), though can be increased or decreased depending on species-specific reactions to reflex tests. For fish, for example, equilibrium, gill and VOR reflexes are strong indicators by themselves, and may be sufficient as mortality predictors. However, curves can be strengthened by additional reflexes provided they are responsive. Curves may also be supplemented or improved by the addition of other factors to further explain variability, as seen in Walters *et al.* (2022), where injury and salinity were added to the vitality-survival curve (see also following section on contextual factors).

Beyond reflexes, specific gear types can cause injuries that strongly predict post-release mortality (e.g. severe skin or scale loss, deep-hooking leading to bleeding); many fishery researchers therefore include types of macroscopic injury in their assessment tools. Although not vitality indicators *per se*, a severely injured fish may exhibit reflex impairments. Such impairments may not be immediately apparent and could develop over time as opportunistic pathogens infect damaged tissues. To that end, pairing indicator assessments with injury scoring may increase the strength of predictions (Nguyen *et al.,* 2014; Uhlmann *et al.,* 2016; Meeremans *et al.,* 2017). It is our perspective that reflex indicators are likely a stronger indicator of short-term mortality (hours to days) rather than longer term mortality.

Holding individuals before reflex assessments may impact the results of the testing, though it is unclear in which direction. While holding tanks or pens have the potential to induce stress and injury, holding periods may also give impaired fish the opportunity to recover before testing—an opportunity that quickly discarded fish would not receive. Though we found similar correlation of vitality and survival between studies which held individuals and those that did not (78% and 82%), we maintain that reflexes should be assessed quickly for best results.

### Contextual factors

Vitality indicators should ideally be calibrated against additional factors because physiology is closely linked to the environment (Fry, 1947). Stressor severity, in particular, can be expected to have a strong influence on an animal’s response (Raby *et al.,* 2013). A number of studies identified that vitality indicators were often sensitive to study treatments, where more stressful treatments such as prolonged air exposure resulted in greater loss of vitality. Some studies have used these results to suggest changes to handling procedures in order to minimize stress and increase post-release survival (e.g. Lennox *et al.*, 2015). Barkley and Cadrin (2012) noted that longer air exposure duration increased RAMP scores and was predictive of post-release mortality; the conclusion was that post-release mortality could therefore be reduced if air exposure duration was reduced. Likewise, Campbell *et al.* (2010a, 2010b) found that a reflex impairment and barotrauma index was predictive of reduced swimming speed and simulated predator approach distance for red snapper following simulated capture. In barracuda, fisheries-related stress resulted in reflex impairment which in turn affected post-release refuge seeking behaviour (Brownscombe *et al.*, 2014), a finding also observed by Cooke *et al.* (2014) for Spanish Flag Snapper (*Lutjanus carponotatus*) on the Great Barrier Reef. Even if vitality indicators were not predictive of mortality (e.g. in experiments where zero mortality actually occurs or where there is simply no relationship), vitality indicators tended to be responsive to more ‘stressful’ experimental treatments (Nguyen *et al.*, 2014). For example, Donaldson *et al.* (2012) identified that the severity of experimental net entanglement increased the risk of injury and reflex impairment; the latter was correlated to post-release mortality for maturing sockeye salmon.

We caution that the shape of a vitality–survival response curve could change dramatically when examining a different species, gear-type or even for the same species/gear combination if changes to some abiotic or biotic factors are substantial enough (e.g. a large change in temperature, body condition, sex ratio or pre-capture disease status of the discards, or differences in predator burden; Patterson *et al.*, 2017a). In Raby *et al.* (2013), chum and pink salmon at spawning grounds were extremely resilient to capture-related stressors that likely would have caused much more mortality earlier in the migration at higher water temperatures. For this reason, using vitality alone to generate a number that can be used as a mortality estimate (which is often what fisheries managers require) is fraught with uncertainty. Environmental contexts can have synergistic or antagonistic interactions with reflex responses, e.g., the presence of predators or stressful water temperatures. As a minimum prerequisite, we would suggest that vitality indices only be used to develop mortality estimates if a relationship between the same vitality assessments has been validated as a mortality predictor with survival in a similar species and fishery. From there, vitality scores can be used to assign mortality risk to discards but only in a relative sense—using those data to generate an exact mortality estimate would require expert opinion, the integration of prior knowledge about factors leading to mortality, information on mortality rates of similar species in the same fishery (if available), and perhaps consultation with a decision scientist and use of a ‘fuzzy logic’ or Bayesian network approach (Varis and Kuikka, 1999; Jarre *et al.*, 2008). To our knowledge, the use of expert input with a fuzzy logic or Bayesian network approaches has yet to be applied to the problem of estimating release mortality estimates (but see ICES, 2015).

### Research and development: a framework

**Figure 3:**
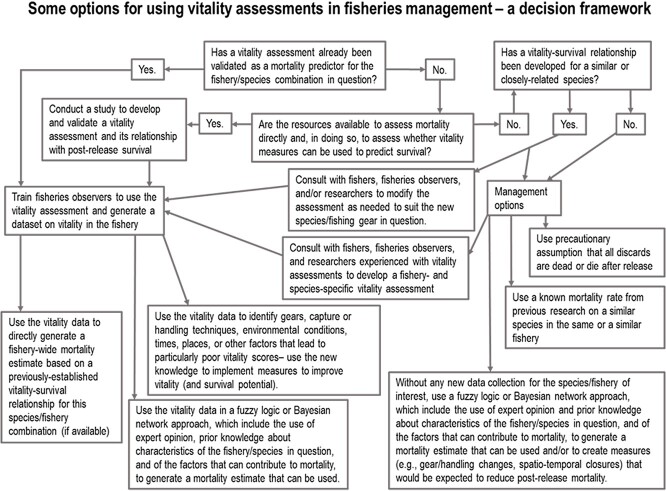
A decision framework for applying vitality indicators to fisheries management.

**Figure 4:**
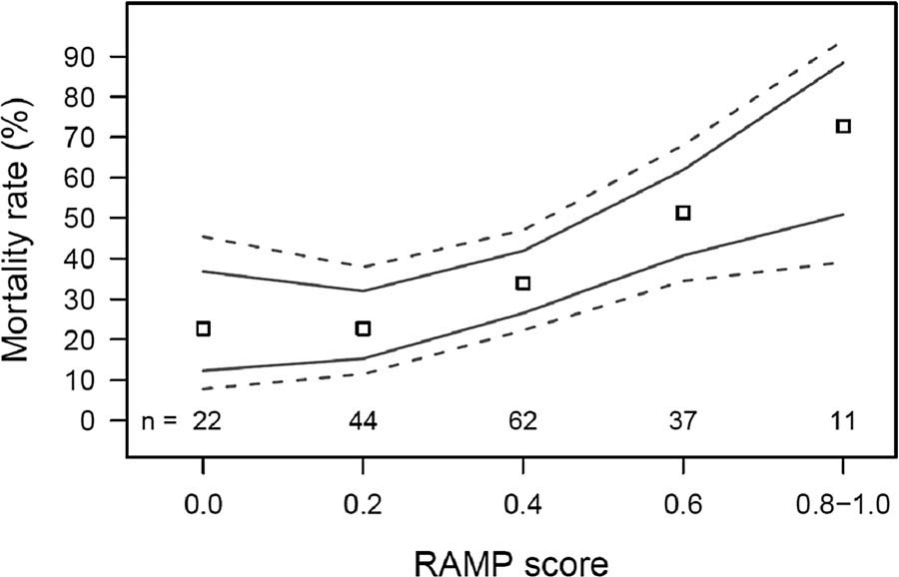
Mean (symbols) and upper and lower 75% (solid lines) and 95% (dashed lines) binomial confidence intervals for post-release mortality rate in relation to Reflex Action Mortality Predictor (RAMP score) in coho salmon released from an aboriginal beach seine fishery (data from Raby *et al.*, 2014). Higher RAMP score indicates that the fish is showing greater signs of reflex impairment (= proportion of reflexes absent). Confidence intervals were calculated using the Clopper–Pearson Exact method.

For a species or fishery for which a vitality–survival relationship has not been developed, it is much less clear how fisheries management would use vitality data and were it to be collected by at-sea observers or via other means (see Fig. 3 for a framework). If a vitality–survival curve has already been validated for the same fishery in a closely related species, or for the same species and in a similar fishery, then the same vitality assessment could be applied and the resultant data used much in the same was as is outlined above, except that the data would have to be weighted more cautiously. For instance, the upper or lower confidence intervals of the original vitality–survival curve (see Fig. 4) could be applied to the new fishery/species depending on whether conservative or precautionary assumptions are warranted based on expert opinion and the judgement of the fishery managers. In the case of a critically endangered species for which there is a vitality–survival curve for a close relative, an option would be to use the upper 75 or 95% confidence interval values for mortality at each vitality score (Fig. 4) when generating an estimate, rather than using the mean. Decisions about adopting more or less conservative estimates for use in management are the responsibility of managers so here we are simply presenting exemplar options. Patterson *et al.* (2017a) serves as a useful template for thinking about how to develop and use vitality indicator for predicting mortality in the absence of validation. It is always important to acknowledge that the use of surrogate species for understanding physiology is controversial (Cooke *et al.*, 2013) and should be approached with ample caution. In urgent cases, expert opinion could be used to judge whether the new fishery/species combination being evaluated is likely to experience higher or lower mortality than in the original study in which the relationship was developed (e.g. depending on differences in gear, water temperature, handling techniques or characteristics of the animals), and this judgement used to assign either upper or lower confidence interval values to mortality estimates (or the mean). Knowledge of the system where one is working can also be relevant for understanding the likelihood that vitality indicators would be predictive of fate. For example, for small fish in predator-rich environments, the inability to maintain equilibrium or burst would make individuals highly susceptible to predation upon release.

### Operationalization

In cases where a fishery and species-specific vitality index is validated as a survival predictor (e.g. Raby *et al.*, 2012; Stoner, 2012), fisheries managers can take up the option of having observers assess a subset of the fishery’s discards (as well as the environmental and operational conditions they were caught in), and use those data to a) generate a rigorous, fishery-wide mortality estimate (i.e. a large sample size with wide spatiotemporal coverage) (See Patterson *et al.*, 2017b) or create guidelines or mandatory regulations relating to handling practices that are shown to improve vitality and therefore increase post-release survivorship. In many cases, it is likely already obvious to the at-sea observers, fishers and managers what changes to fishing or handling techniques could be used to improve survival. However, vitality–survival relationships could be used to estimate the benefits of such changes and in doing so, provide the evidence needed to enter into a cost–benefit analysis of such changes (e.g. where the change in question would incur a cost to the fishery).

### Limitations

There are a number of limitations and challenges associated with the use of vitality indicators (Table 2). When attempting to predict post-release mortality, the interaction among vitality measures should also be considered (Breen and Catchpole, 2021). Wounds inflicted during capture are a major source of mortality for released fish and escapees (Baker and Schindler*,* 2009; Gilman *et al.,* 2013). In a study quantifying the relative effects and interactions between air exposure and injury in sockeye salmon, vitality scores indicated sublethal effects from air exposure treatment but not from an additional and brief gillnet entanglement that elicited minimal injury, even though the latter fish may have experienced added stress based on blood plasma indices (Nguyen *et al.*, 2014). Ongoing research on Pacific salmon in both coastal waters and in-river suggests that in some cases the injury components of a vitality assessment can be more important predictors of post-release mortality than the reflex impairment components, especially with very damaging gear types such as gill nets (Cook *et al.*, 2018a, 2018b). In coho salmon captured by purse seine and held in an on-board tank for 24 h after capture, net injuries and loss of equilibrium were both important precursors to mortality (Raby *et al.*, 2015). Occurrence of capture-induced injury is likely to be particularly relevant to mortality in return migrant Pacific salmon as immune responses disappear through migration (Dolan *et al.,* 2016), limiting capacity for wound healing and increasing susceptibility to pathogen infection and disease (Miller *et al.*, 2014). Therefore, in Pacific salmon, the most comprehensive approach to using vitality to predict mortality combines assessments of reflex impairment and injury because both have the potential to exacerbate natural senescence/disease processes. Nevertheless, there are important differences among fishing gears and locations such that in some situations, injury may be a minor factor or the injuries that need to be assessed may be completely different (e.g. hooking injuries vs. netting injuries). As a result, the injury component of a vitality assessment needs to be tailored to a specific fishing gear, whereas within a species, the same set of reflex assessments should be transferable across fishing gear types (e.g. righting reflex, tail grab response).

**Table 2:** Challenges and limitations of using vitality indicators experimentally and applying them to fisheries and fisheries management, along with associated possible solutions and relevant citations

**Challenge/limitation**	**Description**	**Possible solution**	**Example reference**
Determining which metrics are most effective to predict vitality and the relative contribution of each	A number of vitality indicators have been proposed but some are more effective than others and some may be context-specific	Validation studies that are species/taxa-specific	Davis and Ottmar (2006)
Monitoring post-release survival	In order to conduct validation studies and also to test effectiveness in the field, it is essential to have the ability to effectively monitor post-release survival. Laboratory studies are possible (e.g. Humborstad *et al.,* 2009) but they often lack realism	Telemetry Lab/holding studies.	Raby *et al.* (2012); Donaldson *et al.* (2012)
Understanding temporal relevance of vitality indicators	It is unclear the time frame over which vitality indicators are relevant. For example, it is difficult to imagine that reflex impairments observed in an animal at time of release would be predictive of fate, say 1 year later	Additional research needed to better understand the ways in which reflex indicators are relevant over various time scales	
Lack of understanding of the mechanisms underlying vitality indicators	Most of the reflexes or other indicators discussed here are external manifestations of underlying physiological and neuronal disfunction. Better understanding the mechanisms and biological pathways that lead to such impairments and contribute to fish death (see Holder et al. 2023) could inform the selection of vitality indicators and their application	Additional mechanistic research to understand the physiological and neurological basis for various impairments	
Accounting for observer effects	Observer subjectivity in fish vitality scoring can affect the precision and accuracy vitality indicator scoring analysis	Statistical approaches, such as a mixed-effects multinomial proportional-odds model can be used for modelling ordinal vitality data also to account for observer subjectivity	Benoit *et al.* (2010)
Accounting for co-variates	How to factor in co-variates that could influence survival, including different components of vitality indicators and understanding which metric is more effective at predicting mortality (e.g. reflex impairment vs injury) and extrinsic factors such as environmental conditions.	Experimental approaches designed to control co-variates.	Nguyen *et al.* (2014)
Measuring reflexes on large animals	Invertebrates and smaller turtles and fish can be handled with relative ease which enables reflex assessment. Working with larger animals (e.g. adult sea turtles, sharks, sturgeon) introduces challenges, especially if reflexes need to be measured alongside a boat	Some reflex assessments can be done without having to bring animals about vessels or onto shore	McLean *et al.* (2016)

(Continued)

**Table 2:** Continued

**Challenge/limitation**	**Description**	**Possible solution**	**Example reference**
Maintaining consistency when measuring vitality indicators	Accuracy is essential when determining whether a vitality indicator is positive or negative	Practice is required. Use a single observer if possible. If more than one observer is required, all observers must be carefully trained. More research needed on the strength/importance of observer effects for vitality data	Meeremans *et al.* (2017)
Training vitality raters effectively	In order to put vitality indicators into practice, fisheries observers, technicians, or fishers themselves must be trained. Once trained, there is relatively good agreement among raters (Uhlmann *et al.,* 2020)	Ensure only the simplest and most important vitality indicators are kept in the assessment. Researchers can work with fishers to provide training on-board vessels or in workshops	Meeremans *et al.* (2017); Uhlmann *et al.* (2020)
Ensure that selected vitality indicators are not inherently subjective	Research has revealed that if an indicator is particularly subjective then there tends to be greater variation in whether it is rated as present or absent (Uhlmann *et al.*, 2020)	Ensure that vitality indicators are tested and focus on selecting those for which the vitality indicators are not subjective	Uhlmann *et al.* (2020)
Integrating vitality indicators into fisheries management	There are a number of steps required in order to integrate vitality indicators into fisheries management (Fig. 3)	Sufficient data are required before recommendations can be made and careful consultations with managers are required to ensure that researchers are providing managers with the data they require to integrate vitality indicators into their management frameworks	

Vitality assessments are designed to be inexpensive and accessible, but there is some subjectivity involved in assessments that can affect their utility. Meeremans *et al.* (2017) assessed the variation in scoring for those rating levels of impairment in European plaice. The study revealed modest differences among raters but notably those differences did not significantly influence the relationship between impairment and predicted survival. More recently, Uhlmann *et al.* (2020) assessed the influence of raters on scoring vitality indicators for common sole and found remarkable consistency except for some of the more subjective indices. Conducting tests requires minimal training, but standardization among observers is still important to ensure consistent scoring and ease of interpretation (Meeremans *et al.*, 2017; Uhlmann *et al.*, 2020). We anticipate that new indices will continue to be tested and validated for a variety of species and emphasize the importance of using simple indicators that are easy to conduct, can be accomplished without the use of specialized tools, can take place rapidly, and require minimal interpretation by the observer. For example, Morfin *et al.* (2019) tested an alternative technique, comparing the use of ordinal categories (e.g. excellent, good, poor, moribund) to create a semi-quantitative assessment index (SQA) with RAMP and injury scores, finding that the SQA was highly correlated with RAMP. In RAMP testing, however, we believe that pass/fail metrics for responses are ideal (rather than scoring on a scale to five or ten) but it should be clear how long to take to assess the indicator and how to interpret half responses when they occur (Uhlmann *et al.*, 2020). It is also important to protect against expectation bias (e.g. where animals exposed to a stressor such as air exposure for a longer period would be expected to have more impairments and thus judged accordingly even if there were no differences from non-stressed animals) although the only study on the topic thus far (i.e. Uhlmann *et al.*, 2020) suggests it was not an issue with common sole. Costs of using these indicators is low, but for commercial operations it may take time that is costly to fishers. Moreover, the cost of additional handling or time taken to test each fish may prolong holding or handling for fish that are waiting to be discarded and potentially affect testing results (Rodgveller *et al.,* 2022). Releasing a batch and holding back a small number for testing to extrapolate responses to the batch could be useful in commercial fisheries.

Integration of new scientific evidence or methods into fisheries management is slow and cautious (Young *et al.*, 2016). However, vitality assessments have been used by Fisheries and Oceans Canada (DFO) in both Atlantic and Pacific Ocean fisheries (e.g. Farrell *et al.,* 2001; Benoit *et al.,* 2010). Perhaps the area showing the most potential for application to fisheries management is in improving estimates of post-release mortality used to manage by-catch in commercial fisheries that release non-target species alive. Although it should be carefully communicated that estimates generated using vitality measures alone are inherently less reliable than those obtained from direct observation (e.g. telemetry tracking, net pen holding experiments), the ease of use allows for comparisons among capture-related factors (e.g. gear types, maturation status, population, capture location, etc.) and triage of relative mortality risk for individual fish, especially within a fishery where a vitality–survival curve has already been validated.

## Case study: application of vitality to management of pacific salmon fisheries

In this case study, we aim to summarize literature that used vitality indicators in Pacific salmon and discuss their potential application to management of Fraser River salmon fisheries, which are managed by the DFO. The anadromous return migrations of Pacific salmon from marine feeding areas to natal freshwater breeding areas are cyclical, resulting in dense aggregations of fish that are predictable in time and space and make them especially vulnerable to capture (McDowall, 1999). Pacific salmon are targets of multi-sector fisheries (i.e. commercial, recreational and First Nations) and because some level of live release occurs in most of these fisheries, Pacific salmon are an opportune model for studying release fate. Populations and species can co-migrate in coastal waters and through large rivers before diverging to natal streams. Therefore, most salmon fisheries capture a mix of populations and species, only some of which are sufficiently abundant to sustain harvest (Healey, 2009). In order to protect more vulnerable species or populations, Pacific salmon fisheries in British Columbia have adopted more selective fishing practices that includes spatiotemporal closures, gear restrictions and live release of non-target species (Department of Fisheries and Oceans, 2001). A mortality estimate is often assigned to each species released from each gear type within each geographic fishing area. However, the scientific evidence supporting these mortality estimates is lacking in many cases, and the accuracy of the numbers has been questioned by both stakeholders and academics (Butler, 2005; Walters *et al.*, 2008; Raby *et al.*, 2014, 2015). The resources required to conduct fishery-specific assessments of post-release mortality are extensive, such that it is unrealistic to conduct sufficient studies that provide mortality estimates for each species, fishing area and fishing gear combination (>1000 combinations) is unrealistic. As a result, there is a clear need to establish a rapid and inexpensive means to assess the risk of post-release mortality for fisheries or species of concern and to identify the aspects of capture that could be modified to reduce mortality risk in released fish.

There are several examples of vitality research that have informed, or show potential to inform, the management of Pacific salmon fisheries across a range of sectors (i.e. Indigenous, commercial, and recreational). Tissue biopsies enable researchers to accurately determine an individual fish’s stock complex with molecular genetics and the spawning area that the fish is migrating towards (Beacham *et al.,* 2004), which enables the determination of individual survival to spawn and hence can validate observed measures of vitality (Donaldson *et al.,* 2008). Raby *et al.* (2012) provided a comparison of telemetry-based survival analyses with RAMP scores using wild coho salmon (*O. kisutch*) released from in-river beach seine fisheries. Coho salmon that failed to reach natal sub watersheds exhibited significantly greater reflex impairment scores than did successful migrants; with only one reflex impaired, survival was 93% but this dropped to 44% when three of five reflexes were impaired (Raby *et al.,* 2014).

Measurements of vitality have been used to provide evidence on the effects of different methods of handling and capture in Pacific salmon fisheries. For example, vitality measures have emphasized the increased vulnerability to capture stress at warm temperatures (Gale *et al.*, 2014) as well as the importance of reducing air exposure (Gale *et al.*, 2014; Robinson *et al.,* 2015; Cook *et al.*, 2018a) and handling times (Raby *et al.*, 2014; Cook *et al.*, 2018a). Vitality indicators measured by Gale *et al*. (2014; ventilation rate and duration of equilibrium loss) corresponded with treatment severity and were significant mortality predictors even though treatment did not consistently have an effect on mortality in the holding study. By encompassing the whole-animal response to the capture stressor, vitality indices can identify differential effects among treatments observable immediately following capture that may not necessarily be reflected in mortality patterns (see another example in Donaldson *et al.*, 2012). In the wild, a suite of fisheries-independent factors could contribute to mortality after release (e.g. high water temperatures, natural barriers, pathogen exposure), which is why measures of vitality can be informative of the stress of fisheries capture even if post-release mortality patterns are less clear-cut.

While vitality indicators hold promise to be used as a tool in Pacific salmon fisheries management, inconsistencies have been observed and in some cases vitality indicators have failed to predict mortality. Nguyen *et al.* (2014) measured both injury and reflex impairment following simulations of fisheries capture in freshwater using sockeye salmon, neither of which was statistically linked to mortality rates in released telemetry-tagged fish. Yet, in the marine environment using chum salmon, Cook *et al.* (2018a) observed interactions between injury and vitality indicators with a number of statistically significant effects. In another telemetry study by Robinson *et al.* (2015), mean RAMP scores were very similar among unsuccessful and successful migrants. Raby *et al.* (2015) paired holding studies with telemetry tagging to observe post-release mortality from purse seine fisheries and reflex impairment only predicted mortality in holding studies, leading authors to the conclusion that small sample sizes in the tagging study limited power to derive a RAMP-mortality relationship. In another example, vitality measured in sockeye salmon corresponded to capture treatment severity in one study population but not the other (Donaldson *et al.*, 2012) yet for several populations of coho salmon captured in the ocean, no population-specific variation in the utility of vitality metrics were observed (i.e. there were significant associations between reflex impairment and mortality for all populations; Cook *et al.*, 2018b). Therefore, although vitality measures are showing promise to inform fisheries management, further research is required to understand why these inconsistencies might exist. In some of these studies (Donaldson *et al.*, 2012; Nguyen *et al.,* 2014; Robinson *et al.,* 2015), a lack of concordance between RAMP scores and mortality rates also coincided with a lack of differentiation among experimental capture treatments in post-release mortality. Thus, given that differential stressor severity (i.e. different netting and air exposure treatments) did not create different levels of post-release mortality in these studies, it is not surprising that the among-treatment differences in RAMP scores that *did* differ among treatments were not predictive of mortality. Indeed, RAMP and other vitality indicators are likely only of value in fisheries where two conditions are true: (i) the severity of the capture stressor spans a broad range, and (ii) the likelihood of post-release mortality increases with more severe levels of capture stress (the response to which can be quantified using vitality indicators). For example, in a scenario where the likelihood of an individual fish dying after release is relatively independent of the nature of the capture stressor (e.g. air exposure durations, gear types, handling times) because of (for example) a high period of natural or predation mortality, or adverse environmental conditions, it may be difficult to predict individual post-release mortality (using any metric).

## Conclusions

Vitality indicators hold promise as a low-cost and easily implemented suite of tools to predict fisheries post-release mortality. Operationalizing these tools in fisheries is challenging and requires not only biological assessment, but social science research to understand acceptability among fishers and management agencies. Modelling post-release mortality can benefit from more accurate data that can be estimated based on release condition of discarded fish rather than fixed numbers sometimes input into models. Indeed, there is an ongoing push to integrate vitality measures into fisheries observer programs in some places, including in European groundfish fisheries (ICES, 2014), and for other fisheries (e.g. Pacific salmon). As with many ecological interventions, the question is always how much evidence is enough to justify implementation. In some fisheries, there is probably sufficient data to warrant integrating these tools. In others, specific validations should be conducted. Cost–benefit analyses may also be warranted given the additional time it takes fishers to make and document assessments, forward data to data users including management agencies with oversight responsibility, and for users to interpret and model the results. Utilizing existing data and developing models may be one approach to fill data gaps combined with simple empirical validations. Once validated, vitality indicator assessments have the potential to be efficient tools for fisheries research and monitoring that should continue to increase in relevance for aquatic species exposed to fishing pressure. We acknowledge continued need for additional research related to the operationalization of these tools (like the studies of inter-rater variability; e.g. Uhlmann *et al.,* 2020), field validation (e.g. using telemetry; Raby *et al.,* 2012) and understanding the mechanistic basis and biological pathways underpinning vitality indicator impairments.

## Supplementary Material

Web_Material_coae034

## Data Availability

Data will be made available upon reasonable request.
